# Influence of preoperative urine culture and bacterial species on urogenital sepsis after ureteral flexible lithotripsy in patients with upper urinary tract stones

**DOI:** 10.3389/fmed.2024.1393734

**Published:** 2024-05-03

**Authors:** Leibo Wang, Xianzhe Yu, Zuze Qiu, Puyu Liu, Wu Tian, Wei He, Yulin Pan, Feng Xu, Zhuangding Cen, Yang Ou, Daobing Li

**Affiliations:** ^1^Department of Surgery, Beijing Jishuitan Hospital Guizhou Hospital, Guiyang, China; ^2^Department of Urology, Affiliated Hospital of Zunyi Medical University, Zunyi, China; ^3^Department of Gastrointestinal Surgery, Chengdu Second People’s Hospital, Chengdu, China; ^4^Department of Pathology, Affiliated Hospital of Zunyi Medical University, Zunyi, China; ^5^Hangzhou Litchi Medical Beauty Clinic, Hangzhou, China

**Keywords:** ureteral flexible lithotripsy, urine culture, *Escherichia coli*, urosepsis, urinary tract stones

## Abstract

**Objective:**

This retrospective study aims to identify risk factors for urogenic sepsis in patients with upper urinary tract stones following ureteral flexible lithotripsy (FURL). Additionally, we analyze the clinical characteristics of bacterial infections post-surgery.

**Methods:**

A total of 759 patients who underwent FURL at the Urology Department of Zunyi Medical University were included. Univariate and multivariate Logistic regression analyses were conducted to identify independent risk factors for urogenic sepsis post-FURL. The distribution of bacteria based on preoperative urine cultures was also analyzed. Statistical analysis was performed using R4.2.2 software.

**Results:**

Of the 759 patients, positive preoperative urine culture, urine nitrite positivity, urine white blood cell count (WBC) ≥ 200 cells/μL, residual stones, and neutrophil-to-lymphocyte ratio (NLR) were found to be independent risk factors for urogenic sepsis after FURL. Among the 164 patients with positive preoperative urine cultures, 32 developed urogenic sepsis post-surgery, with 68.75% having positive preoperative cultures. The leading pathogens causing postoperative urogenic sepsis were *Escherichia coli* (*E. coli*), *Enterococcus faecium*, *Proteus mirabilis*, and *Klebsiella pneumoniae*. The probabilities of progression to urogenic sepsis were as follows: *E. coli* 19% (*n* = 12), *Enterococcus faecium* 43% (*n* = 3), *Proteus mirabilis* 33.3% (*n* = 1), and *Klebsiella pneumoniae* 33.3% (*n* = 1). The ages of affected patients were 47.17 ± 13.2, 53.7, 41, and 79 years, respectively. Rates of comorbid diabetes were 36.4, 66.7, 50, 100%, with nitrite positivity rates at 72.7, 33.3, 50, 0%. Ten female patients were infected with *E. coli*, while patients infected with *Klebsiella pneumoniae* had an NLR of 7.62.

**Conclusion:**

Positive preoperative urine culture, urine nitrite positivity, urine WBC ≥ 200 cells/μL, residual stones, and NLR are independent risk factors for urogenic sepsis after FURL. *Escherichia coli* is the predominant pathogen post-FURL, with notable female prevalence and nitrite-positive urine in infections. *Enterococcus faecium* infections are associated with diabetes.

## 1 Introduction

Urolithiasis stands as a prevalent ailment in urology, with the worldwide incidence of urinary stones ranging from 1 to 20%, and specifically within China, it falls between 1 and 5% (1, 2). The global prevalence of urolithiasis has been on the rise, attributed to shifts in dietary patterns, socioeconomic factors, and climatic variations (3). Notably, the recurrence rate within a decade following treatment is notably high, hovering around 50% (4). The escalating trends in urolithiasis pose significant health threats to patients and impose substantial financial burdens. Studies forecast a projected annual increase of $1.24 billion in costs associated with urolithiasis by the year 2030 (5). This pressing issue underscores the urgent need for effective prevention strategies and treatment modalities to mitigate the impact on patient well-being and healthcare expenditures.

In recent decades, the gradual emergence of endoluminal urological techniques has significantly expanded the use of minimally invasive procedures such as percutaneous nephrolithotomy, ureteroscopy, and ureteral flexible scopes. Notably, flexible ureteroscopic lithotripsy has emerged as a safe and effective treatment option for upper urinary tract stones due to its advantages of providing a clear surgical field, minimal invasiveness, and swift recovery (6). The European Association of Urology (EAU) has endorsed this technique as the primary treatment for kidney stones measuring ≤2cm (7). Despite the benefits of these procedures, a spectrum of surgical complications can arise, with urinary sepsis representing one of the most severe, with incidence rates ranging from 0.1 to 4.3% (8). Urinary sepsis is particularly concerning due to its rapid onset, swift progression, and alarmingly high mortality rate, which can reach up to 50% (8).

A positive mid-section urine culture is closely associated with the development of urosepsis following FURL (9–11). The release of bacteria and endotoxins from within the stones during the procedure is a significant factor leading to urinary tract infections, Systemic Inflammatory Response Syndrome (SIRS), and potentially urosepsis (9). The detailed analysis of bacterial profiles holds significant clinical importance for preventing and treating postoperative urogenic sepsis. Therefore, this study aims to investigate the risk factors for urogenic sepsis following ureteral lithotripsy in patients with upper urinary tract stones, with a specific focus on preoperative urine culture results. Additionally, the study aims to analyze the clinical characteristics of various bacteria in patients who develop postoperative urogenic sepsis. The overarching goal is to provide clinicians with valuable insights and guidance for the early prevention and effective treatment of postoperative urogenic sepsis subsequent to ureteral lithotripsy using flexible ureteroscopes.

## 2 Materials and methods

### 2.1 Clinical data

Patients diagnosed with kidney stones or upper ureteral stones and admitted to the Affiliated Hospital of Zunyi Medical University between January 2019 and December 2022 were enrolled as study participants. Inclusion Criteria: 1. All cases were confirmed using computed tomography (CT). 2. Patients underwent treatment with ureteral flexible scope lithotripsy for upper urinary tract stones and had complete medical records. Exclusion Criteria: 1. Postoperative sepsis resulting from infections in other organ systems. 2. Patients with immunocompromised conditions such as tumors, blood disorders, or those taking oral immunosuppressive drugs. 3. Individuals with congenital anomalies such as polycystic kidneys, horseshoe kidneys, or pelvic-ureteral junction obstruction. 4. Cases with incomplete or insufficient medical records. The retrospective study was approved by the Ethics Review Board of the Affiliated Hospital of Zunyi Medical University. Informed consent requirements were waived by the Ethics Review Board due to the minimal risks associated with retrospective studies.

All surgeries were performed by attending physicians with extensive experience in the department. The principles of antibiotic use in this study were carried out according to the Chinese Guidelines for the Treatment of Urologic Stones. If preoperative urine culture is negative but there is a urinary tract infection, appropriate antibiotics such as levofloxacin, amikacin, or others should be chosen based on local bacterial spectrum and resistance profiles. The antibiotic should be selected according to the treatment course, which is typically 1 week. During the operation, first-generation, second-generation, and fluoroquinolone drugs are used for prophylaxis. If preoperative urine culture is positive or preoperative urine routine shows positive nitrites the surgeon should choose oral or intravenous antibiotics according to the drug sensitivity results. One week later, if the urine culture results are negative before surgery, the patient can undergo the surgery. If the urine culture remains positive, continue with anti-infection treatment. If preoperative urine culture is negative and there is no obvious urinary tract infection, first-generation, second-generation cephalosporins, and fluoroquinolone drugs are used for infection prophylaxis. If there are no postoperative infectious complications, the total treatment course should be ≤24 h.

### 2.2 Collection of clinical data

Data collection for this study was a collaborative effort involving two researchers specializing in urology. Additionally, the collected data underwent scrutiny by two individuals to ensure its accuracy and validity. The study included various patient characteristics such as age, gender, presence of comorbid hypertension, comorbid diabetes mellitus status, stone location (side and site), stone size, presence of hydronephrosis, preoperative neutrophil count, absolute blood lymphocyte count, Neutrophil-to-Lymphocyte Ratio (NLR: calculated as preoperative absolute neutrophil count divided by blood lymphocyte count), absolute blood leukocyte count before surgery, preoperative urinary leukocyte count, urine nitrite test (NIT), preoperative creatinine levels, results of preoperative urine culture with drug sensitivity, duration of surgery, amount of bleeding during surgery, and presence of residual stone.

### 2.3 Infection criteria

The diagnostic criteria for urogenic sepsis align with the guidelines set forth by the European Society of Urology in 2018 (12). According to these guidelines, urogenic sepsis is diagnosed when a patient presents with a urinary tract infection accompanied by systemic inflammatory response syndrome (13). The confirmation of this diagnosis requires the presence of at least two of the following SIRS criteria: (1) temperature > 38°C or < 36°C; (2) heart rate > 90 beats/min; (3) respiration >20 breaths/min or hyperventilation with PaCO2 < 32 mmHg; (4) blood leukocyte counts >12 × 10^9/L or < 4 × 10^9/L, or immature granulocytes >10%. Additionally, the patient may exhibit concurrent symptoms of urinary tract infection or have a positive blood culture.

### 2.4 Statistical methods

All statistical analyses for this study were performed using R version 4.2.2 statistical software. Continuous variables were expressed as mean ± standard deviation or median with interquartile range, while categorical variables were presented as counts and corresponding percentages. Initial analysis of patient clinicopathological characteristics was conducted using Logistic univariate regression. Variables exhibiting statistically significant differences were subsequently included in a multivariate Logistic regression analysis to identify independent risk factors. The results of the multivariate Logistic regression analysis were then used to screen for these independent risk factors. With *p* < 0.05 for the difference is statistically significant.

## 3 Results

### 3.1 General characteristics of clinical data

A total of 759 cases of postoperative patients undergoing ureteroscopic lithotripsy in the Department of Urology, Affiliated Hospital of Zunyi Medical University, were included in this study. Among them, 32 patients developed urosepticemia after surgery. In the non-urosepticemia group, there were 142 (19.53%) patients with positive urine cultures, 307 (42.23%) patients with surgical times exceeding 90 min, and 205 (28.20%) patients with stones ≥20 mm. In the urosepticemia group, 46.88% (*n* = 15) of patients had positive urinary nitrite, 68.75% (*n* = 22) had urinary leukocytes exceeding 200 cells/μL, and 17 (53.12%) patients had positive urinary nitrite. Detailed general clinical characteristics of the patients are shown in Table 1.

**Table 1:** General clinical characteristics of patients in urosepsis and non-urosepsis groups.

Parameters	Urosepsis (*n* = 32)	Non-Urosepsis (*n* = 727)	*p*-value
Age (%), years			
<60	23 (71.88)	603 (82.94)	0.1693
≥60	9 (28.12)	124 (17.06)	
Gender (%)			
Male	9 (28.12)	420 (57.77)	0.0018
Female	23 (71.88)	307 (42.23)	
Blood loss (%)			
<10 mL	9 (28.12)	147 (20.22)	0.39
≥ 10 mL	23 (71.88)	580 (79.78)	
Side (%)			
Left	16 (50.00)	382 (52.54)	0.9193
Right	16 (50.00)	345 (47.46)	
Urine culture (%)			
Positive	22 (68.75)	142 (19.53)	<0.0001
Negative	10 (31.25)	585 (80.47)	
Diabete (%)			
Yes	6 (18.75)	76 (10.45)	0.2346
No	26 (81.25)	651 (89.55)	
Hydronephrosis (%)			
Yes	3 (9.38)	62 (8.53)	1
No	29 (90.62)	665 (91.47)	
Hypertension (%)			
Yes	10 (31.25)	175 (24.07)	0.4744
No	22 (68.75)	552 (75.93)	
Acute renal insufficiency (%)			
Yes	8 (25.00)	121 (16.64)	0.3216
No	24 (75.00)	606 (83.36)	
Location (%)			
Upper ureteric	4 (12.50)	84 (11.55)	0.5947
Renal	25 (78.12)	526 (72.35)	
both	3 (9.38)	117 (16.09)	
Urine nitrite (%)			
Positive	15 (46.88)	64 (8.80)	<0.0001
Negative	17 (53.12)	663 (91.20)	
Operation time (%), min			
<60 min	7 (21.88)	202 (27.79)	0.7554
60-89 min	10 (31.25)	218 (29.99)	
≥90 min	15 (46.88)	307 (42.23)	
Residual stone (%)			
Yes	6 (18.75)	62 (8.53)	0.0959
No	26 (81.25)	665 (91.47)	
Size (%), mm			
<20	19 (59.38)	522 (71.80)	0.1865
≥20	13 (40.62)	205 (28.20)	
Urine WBC (%),/μL			
<100	7 (21.88)	456 (62.72)	<0.0001
100–199	3 (9.38)	106 (14.58)	
≥200	22 (68.75)	165 (22.70)	
Blood WBC [mean (SD)]	6.960 (3.645)	6.312 (1.951)	0.0803
NLR [mean (SD)]	4.154 (5.708)	2.464 (1.897)	<0.0001

### 3.2 Screening of risk factors for urogenital sepsis following flexible ureteral lithotripsy

The results revealed independent risk factors associated with the development of urogenital sepsis after ureteral flexible lithotripsy to be Urine Culture, Urine Nitrite, Urine White Blood Cell count, Residual Stone, and Neutrophil-to-Lymphocyte Ratio, as depicted in Table 2.

**Table 2:** Results of single-factor and multi-factor logistic regression analysis on urosepsis occurrence in patients after flexible ureteroscopy lithotripsy.

Variables	Univariable analysis		Multivariable analysis	
	OR (95% CI) P		OR (95% CI) P	
**Age**				
41–60				
≥60	1.9 (0.86–4.21)	0.11		
**Gender**				
Male				
Female	3.5 (1.6–7.66)	<0.001	2.25 (0.94–5.37)	0.0681
**Blood loss**				
<10 mL	1.54 (0.7–3.41)	0.28		
≥10 mL				
**Side**				
Left	1.11 (0.55–2.25)	0.78		
Right				
Urine culture	9.06 (4.2–19.57)	<0.001	5.35 (2.2–12.99)	<0.001
Positive				
Negative				
**Diabete**				
Yes	1.98 (0.79–4.95)	0.15		
No				
**Hydronephrosis**				
Yes	1.11 (0.33–3.75)	0.87		
No				
**Hypertension**				
Yes	1.43 (0.67–3.09)	0.36		
No				
**Renal insufficiency**				
Yes	1.67 (0.73–3.8)	0.22		
No				
**Location**				
Upper ureteric	1 (0.34–2.94)	1		
Renal	0.54 (0.34–2.94)	0.43		
**Both**				
Urine nitrite				
Positive	9.14 (4.36–19.16)	<0.001	2.74 (1.12–6.7)	0.027
Negative				
**Operation time**				
<60 min				
60-89 min	1.32 (0.49–3.54)	0.58		
≥90 min	1.41 (0.56–3.52)	0.46		
**Residual stone**				
Yes	2.48 (0.98–6.24)	0.05	4.66 (2.2–9.87)	<0.001
No				
**Size**				
<20 mm				
≥20 mm	1.74 (0.84–3.59)	0.13		
**Urine WBC**				
<100				
100–199	1.84 (0.47–7.25)	0.38	1.2 (0.27–5.23)	0.8122
≥200	8.69 (3.64–20.71)	<0.001	3.84 (1.39–10.64)	0.0097
Blood WBC	1.13 (0.98–1.29)	0.08		
Neutrophil	1.18 (1.04–1.34)	0.01	1.05 (0.85–1.3)	0.6211
Lymphocytes	0.58 (0.31–1.06)	0.08		
NLR	1.16 (1.06–1.27)	<0.001	1.2 (1.04–1.39)	0.0137

### 3.3 Bacterial species and clinical features identified in urine cultures causing postoperative urogenic septicemia

Out of 759 patients, 164 had positive preoperative urine cultures. Among the 32 patients with urogenic sepsis, 22 (68.75%) had positive preoperative urine cultures, three of whom had multiple bacterial infections. The bacterial species and associated statistical analysis are detailed in Table 3. The most common causative organisms were *Escherichia coli*, followed by *Enterococcus faecalis* and *Gardnerella vaginalis*.

**Table 3:** Distribution of pathogenic bacteria in patients with positive mid-stream urine cultures.

Bacteria	Case, *n* (%)	Sepsis
*Escherichia coli (E. coli)*	63 (34.4%)	12
*Enterococcus faecalis*	10 (6.1%)	0
*Gardnerella vaginalis*	8 (4.9%)	0
*Enterococcus faecium*	7 (4.3%)	3
*Proteus mirabilis*	6 (3.7%)	2
*Klebsiella pnenmoniae*	3 (1.8%)	1
*Corynebacterium glucuronolyticum*	3 (1.8%)	0
*Pseudomonas aeruginosa*	3 (1.8%)	0
*Staphylococcus epidermidis*	3 (1.8%)	0
*Group B streptococcus*	2 (1.2%)	0
*Streptococcus mitis*	2 (1.2%)	0
*Yeast-like fungus*	2 (1.2%)	0
*Proteus vulgaris*	2 (1.2%)	0
*Citrobacter freundii*	1 (0.6%)	0
*Lactobacillus crispatus*	1 (0.6%)	0
*Staphylococcus lugdunensis*	1 (0.6%)	0
*Proteus pengii*	1 (0.6%)	1
*Hemolytic staphylococcus*	1 (0.6%)	0
*Sphingomonas paucimobilis*	1 (0.6%)	0
*Corynebacterium amyceticum*	1 (0.6%)	0
*streptococcus anginosus*	1 (0.6%)	0
*Burkholderia cepacia*	1 (0.6%)	0
*Multiple bacteria*	40 (27.4%)	3

The probability of progression to urogenic sepsis in patients with *E. coli* infection was 19% (*n* = 12). Among patients with *Enterococcus faecium* infection, three out of seven (43%) progressed to urogenic sepsis, two out of six with *Proteus mirabilis* (*PM*) infection, and one out of three with *Klebsiella pneumoniae* infection. Clinical features of ureterogenic septicemia following ureteral flexible lithotripsy are outlined in Table 4.

**Table 4:** Clinical characteristics of various bacteria causing urogenic sepsis following FURL surgery.

Parameters	*Escherichia coli (E. coli)*	*Enterococcus faecium*	*Proteus mirabilis*	*Klebsiella pnenmoniae*
Age, years	47.17 ± 13.2	53.7	41	79
Gender				
Male, *n*	2	3	2	1
Female, *n*	10	0	0	0
Rate of diabetes, %	36.4	66.7	50	100
Progression rate to urosepsis, %	19	43	33.3	33.3
Nitrite positive rate, %	72.7	33.3	50	0
NLR	3.32 ± 3.6	3.78	1.6	7.62

## 4 Discussion

Despite advancements in urologic laparoscopic techniques and the evolution of lithotripsy and stone extraction equipment, FURL remains associated with postoperative infections, a prevalent complication (11, 14). Urogenic sepsis, as the most severe perioperative complication of FURL, continues to pose challenges for urologists in clinical practice. In this retrospective study, we identified Urine Culture, Urine Nitrite, Urine White Blood Cell count, Residual Stone, and Neutrophil-to-Lymphocyte Ratio as independent risk factors for urogenic sepsis after FURL, using univariate and multivariate logistic regression analyses. Furthermore, we conducted an analysis of bacterial distribution based on preoperative urine culture results, revealing *Escherichia coli, Enterococcus faecalis*, and *Gardnerella vaginalis* as the most common bacteria causing urinary tract infections. Understanding these prevalent urinary tract bacteria and their corresponding infection characteristics can aid in clinical diagnosis and treatment strategies.

The present study confirms that Urine culture stands as a significant risk factor for urogenic sepsis development following FURL, aligning with previous research findings (9, 10, 15, 16). Mi et al. (9) reported that 16 of 21 patients (76.2%) in the SIRS group exhibited positive preoperative urine cultures, with these results significantly correlating with postoperative SIRS development. Similarly, James et al. (17) in a retrospective analysis of 462 patients treated for stones via ureteroscopy, noted a significant association between positive mid-stream urine cultures and postoperative urinary sepsis findings, despite all patients receiving appropriate preoperative antibiotic therapy. Senocak et al. (16) in a retrospective analysis of 492 patients undergoing ureteral flexible lithotripsy, found an 8.5% incidence of postoperative infectious complications. Of the 59 patients (12%) with positive preoperative urine cultures, 19 patients (32.2%) were identified with multidrug-resistant (MDR) isolates from these cultures.

Urinary tract infections (UTIs) should be adequately treated with anti-infective therapy prior to various urologic surgeries, a consensus among major urologic guidelines. In this study, we adhered to the principles of antibiotic use outlined in the Chinese guidelines for urologic stone treatment, administering anti-infective therapy to all patients with positive preoperative urine cultures and evidence of UTIs. Despite this, 32 out of 164 patients with positive preoperative urine cultures developed urogenital sepsis, resulting in an incidence rate of 19.5%. Gutierrez et al. (9, 18) found in their study that even with the administration of broad-spectrum antibiotics to urine-negative patients or sensitive antibiotics to those with positive urine cultures, postoperative Systemic Inflammatory Response Syndrome following flexible ureteroscopic lithotripsy remained inevitable. Patients with gram-negative bacilli in their urine cultures were noted to be more prone to postoperative hyperthermia compared to those with gram-positive bacilli. It has been suggested that the biofilm formed in stones presents a challenge for antibiotics to effectively eliminate bacteria (9, 19). Despite patients with positive preoperative urine cultures exhibiting negative secondary urine cultures after antibiotic treatment and prior to surgery, the presence of bacterial endotoxins within stones complicates antibiotic penetration. Furthermore, significant endotoxin release during lithotripsy increases infection risks, potentially leading to systemic inflammatory responses. Some studies (10) suggest that early antibiotic treatment could be effective in reducing the incidence of SIRS. Considering that results from urine culture sensitivity tests typically take at least 48 hours (20), understanding common urinary tract bacteria and their respective infection characteristics becomes crucial. This understanding aids in the selection of appropriate empirical antibiotic treatment while awaiting urine culture results.

The primary bacteria identified in preoperative urine cultures in this study included *Escherichia coli*, *Enterococcus faecalis*, *Gardnerella vaginalis*, *Proteus mirabilis*, and *Klebsiella pneumoniae*. Among these, *Escherichia coli* ranked highest at 34.4%, consistent with findings from previous studies (21, 22). Cagri et al. (16) reported similar results in their study on risk factors for infectious complications following flexible ureteroscopic lithotripsy, with *Escherichia coli* and *Enterococci* being the predominant pathogens. In our study, *Escherichia coli* infection was notably associated with predominantly female patients and positive urinary nitrites. Furthermore, *Escherichia coli* positivity exhibited a significant association with postoperative urogenic sepsis after ureteral flexible lithotripsy. Patients positive for *Enterococcus faecalis*, *Proteus mirabilis*, and *Klebsiella pneumoniae* were also more likely to develop postoperative urogenic sepsis compared to *Escherichia coli* positivity (43, 33.3, and 33.3% vs. 19%). However, statistical analyses regarding postoperative urogenic sepsis were not obtained for *Enterococcus faecalis* and *Gardnerella vaginalis*, likely due to the small sample size. Studies suggest (21) that one-third of community-acquired urinary tract infections caused by *Escherichia coli* are attributed to ultrawide-spectrum β-lactamase (ESBL) strains, highly resistant to commonly used community antibiotics, thus limiting treatment effectiveness. Another study has shown (23) that the expression of the BLA CTX-M-14 gene in *Escherichia coli* leads to increased resistance, further restricting the empirical use of cephalosporins and fluoroquinolones in urinary tract infection treatment.

Enterococci are commonly identified as pathogens in urinary tract infections (24), with some studies suggesting that they have become the primary causative agent of such infections (22, 25). The main culprits among enterococci are *Enterococcus faecalis* And*enterococcus faecium*, which are generally less virulent compared to other strains (22). In our study, *Escherichia coli* remained the predominant infection, with urogenic sepsis occurring in 3 out of 7 patients positive for *Enterococcus faecium*. Interestingly, no cases of urogenic sepsis were observed in the 10 patients with *Enterococcus faecalis* infections. Additionally, 66.7% of patients with *Enterococcus faecium* infections also had comorbid diabetes mellitus, highlighting this as a significant risk factor. Enterococci exhibit a natural resistance to many antibiotics, and there is a rapid increase in acquired resistance, complicating the treatment of urinary tract infections caused by these bacteria (26). Recent reports indicate a global rise in hospital-acquired vancomycin-resistant enterococcal infections, underscoring the potential for serious nosocomial infections (27). Therefore, heightened surveillance of this bacterial group is crucial for the prevention and control of nosocomial infections.

*Proteus mirabilis* and *Klebsiella pneumoniae* are two additional common bacteria found in our study. Studies have noted that (28) *Klebsiella pneumoniae* exhibits a strong adhesive and invasive capability, allowing it to persist in the urethra and resist urinary tract erosion. This resilience is particularly notable in diabetic patients with inadequate glycemic control, where the bacterium shows increased resistance and a tendency for recurrent infections. Furthermore, research has shown that (29, 30) *Klebsiella pneumoniae* has the ability to produce carbapenemases, acquiring resistance to carbapenems. Strains with this characteristic are also resistant to penicillin and cephalosporins, facilitating their transmission. In our study, patients who developed sepsis from *Klebsiella pneumoniae* infection tended to be of advanced age, with comorbid diabetes mellitus, and exhibited high Neutrophil-to-Lymphocyte Ratio, but the results were limited by the sample. *Proteus mirabilis* (31), a Gram-negative rod-shaped bacterium, is a prominent pathogen causing complicated urinary tract infections. Some studies have suggested that (31) catheter-associated urinary tract infections (CAUTI) in particular are the main pathogens. Due to its ureolytic biomineralization, *Proteus mirabilis* can form a crystalline biofilm on the surfaces of indwelling urethral catheters, leading to catheter scaling, blockage, and, often, urinary retention. This can result in ascending urinary tract infections, such as cystitis and pyelonephritis (32). This bacterium commonly coexists with various other members of the microbial community (32). A deeper understanding of *Proteus mirabilis* will aid clinicians in developing optimal strategies to control infections associated with this bacterium.

In addition to positive urine culture, this study identified positive urine nitrite, urine white blood cell count ≥200 cells/μL, residual stone, and Neutrophil-to-Lymphocyte Ratio as independent risk factors for urogenital sepsis following ureteral flexible lithotripsy. The urinary nitrite test and urine WBC count are commonly utilized for diagnosing urinary tract infections. Studies have indicated a high specificity of urinary nitrite for Gram-negative bacterial urinary tract infections (33). In our study, the risk of sepsis was found to be 2.74 times higher in preoperative urinary nitrite-positive patients compared to nitrite-negative patients. Moreover, debris remaining after ureteral flexible lithotripsy may harbor bacteria and endotoxins, which can enter the vasculature through damaged endothelium, potentially leading to infectious complications (34). The Neutrophil-to-Lymphocyte Ratio (35) serves as a straightforward and effective indicator of a patient’s immune status and infection severity. A retrospective study has demonstrated that (36) NLR values may be more effective than blood WBC and neutrophil counts in predicting or diagnosing urinary sepsis.

This study is subject to several limitations. Firstly, being a single-center retrospective study, it may have inherent selection bias and limited included data. A broader, prospective multicenter study is warranted for further analysis in the future. Secondly, the study did not delve into further analysis of bacterial resistance, which will be a focal point of our future research on this topic. Thirdly, intra-pelvic perfusion pressure during ureteral flexible lithotripsy is often linked to infection-related complications, yet this data was not collected in our study due to equipment limitations.

## 5 Conclusion

In conclusion, this study identified Urine culture positivity, Urine nitrite, Urine WBC count ≥200 cells/μL, Residual stone presence, and NLR as independent risk factors for postoperative urogenic sepsis following FURL. Additionally, the study analyzed the distribution of bacteria based on preoperative urine cultures, with *Escherichia coli* being the most prevalent pathogen associated with postoperative urogenic sepsis. This was followed by *Enterococcus faecalis*, *Proteus mirabilis*, and *Klebsiella pneumoniae*. Key characteristics of *Escherichia coli* infections included a higher prevalence among female patients and positive urine nitrite results. Patients with *Enterococcus faecalis* infections often presented with comorbid diabetes mellitus. Patients with *Klebsiella pneumoniae* infection and sepsis in this study were old, often complicated with diabetes, and had a high NLR. However, due to limited samples, more extensive data will need to be included in the future to further demonstrate this conclusion.

## Data availability statement

The raw data supporting the conclusions of this article will be made available by the authors, without undue reservation.

## Ethics statement

The Ethics Committee of Guizhou Hospital, Beijing Jishuitan Hospital approved this study (20220402). The studies were conducted in accordance with the local legislation and institutional requirements. The ethics committee/institutional review board waived the requirement of written informed consent for participation from the participants or the participants’ legal guardians/next of kin because retrospective studies have extremely low risk.

## Author contributions

LW: Funding acquisition, Methodology, Writing – original draft, Writing – review & editing. XY: Writing – original draft, Methodology. ZQ: Data curation, Writing – original draft. PL: Formal analysis, Writing – original draft, Supervision. WT: Supervision, Writing – original draft. WH: Investigation, Software, Writing – original draft. YP: Methodology, Validation, Writing – review & editing. FX: Project administration, Writing – review & editing. ZC: Methodology, Validation, Writing – original draft. YO: Methodology, Resources, Writing – review & editing. DL: Methodology, Project administration, Software, Supervision, Writing – original draft.
